# A newly discovered biodiversity hotspot of many-plumed moths in the Mount Cameroon area: first report on species diversity, with description of nine new species (Lepidoptera, Alucitidae)

**DOI:** 10.3897/zookeys.777.24729

**Published:** 2018-07-30

**Authors:** Peter Ustjuzhanin, Vasily Kovtunovich, Szabolcs Sáfián, Vincent Maicher, Robert Tropek

**Affiliations:** 1 Altai State University, Lenina 61, Barnaul, 656049, Russia Altai State University Barnaul Russia; 2 Moscow Society of Nature Explorers, Bolshaya Nikitskaya 6, Moscow, RU–103009, Russia Moscow Society of Nature Explorers Moscow Russia; 3 Department of Zoology, Faculty of Science, University of South Bohemia, Branišovská 1760, CZ-37005 České Budějovice, Czechia University of South Bohemia České Budějovice Czech Republic; 4 Biology Centre of the Czech Academy of Sciences, Institute of Entomology, Branišovská 31, CZ-37005 České Budějovice, Czechia Biology Centre of the Czech Academy of Sciences, Institute of Entomology České Budějovice Czech Republic; 5 Department of Ecology, Faculty of Science, Charles University, Viničná 7, CZ-12844 Prague, Czechia Charles University Prague Czech Republic

**Keywords:** Alucitidae, biodiversity, Cameroon, many-plumed moths, Mount Cameroon, new species, new records, tropical rainforest

## Abstract

Fifteen species of many-plumed moths are recorded from the Mount Cameroon area, SW Cameroon, West Africa. Nine species: *Alucitalongipenis* Ustjuzhanin & Kovtunovich, **sp. n.**, *A.lidiya* Ustjuzhanin & Kovtunovich, **sp. n.**, *A.ludmila* Ustjuzhanin & Kovtunovich, **sp. n.**, *A.escobari* Ustjuzhanin & Kovtunovich, **sp. n.**, *A.mischenini* Ustjuzhanin & Kovtunovich, **sp. n.**, *A.fokami* Ustjuzhanin & Kovtunovich, **sp. n.**, *A.janeceki* Ustjuzhanin & Kovtunovich, **sp. n.**, *A.besongi* Ustjuzhanin & Kovtunovich, **sp. n.**, and *A.olga* Ustjuzhanin & Kovtunovich, **sp. n.**, are described as new for science. Four species are recorded as new from Cameroon: *A.acalyptra*, *A.chloracta*, *A.coffeina*, and *A.spicifera*. By these records, the Mount Cameroon area has become the richest known Afrotropical locality for the Alucitidae, highlighting its tremendous value for biodiversity conservation.

## Introduction

Many-plumed moths (Alucitidae) differ from other Lepidoptera by the structure of their wings, which are each split into six lobes, with the exception of the oriental genus *Triscaedecia* Hampson, 1905, which has seven-lobed hind wings. These moths are active at night, and relatively well attracted to light. Most of their diversity is currently known from the Palearctic region, where their caterpillars usually live concealed in plant tissue, some make leafmines, others feed in flowers and buds of various species in the honeysuckle family (Caprifoliaceae). However, the biology of the African many-plumed moths is virtually unstudied.

The taxonomy and distribution of the many-plumed moths are seriously understudied in the Afrotropical Region, despite recent publications of material originating from South Africa, Zimbabwe, and Malawi (Ustjuzhanin and Kovtunovich 2011, 2016). Altogether, 58 species are known from all of the Afrotropics (De Prins and De Prins 2018). Most of the previously described species originate from southern and eastern Africa (Meyrick 1908, 1911, 1913, 1920, Hering 1917, Viette 1958), whereas only 12 species were known to occur in the Guineo-Congolian forest zone, virtually each of them from a few records only (De Prins and De Prins 2018). Nevertheless, this region includes two of the recognised biodiversity hotspots of Africa (Myers et al. 2000) and is thus also expected to harbour many more species of Alucitidae. This is reflected in recent unpublished collections by Sz. Sáfián, who collected several species in Ghana and Liberia.

Since 2014, the last three authors have been coordinating an extensive study of the biodiversity of Lepidoptera on Mount Cameroon, Southwest Province, Cameroon. Its aim was to survey butterfly and moth communities in different altitudinal forest zones on the south-western slopes of the mountain. Originally, Alucitidae were not included in the target groups (which are mainly various groups of Bombycoidea, Noctuoidea, and Geometroidea), but during the first collecting hours, the authors realised how species-rich the family appeared in the study area. Consequently, all Alucitidae specimens were systematically collected during each sampling night. The collected material was sent to the first two authors for identification and further taxonomic work. This paper presents the first results of the study, including descriptions of nine new species, a staggering increase in the known species of Alucitidae from West Africa.

## Materials and methods

### Abbreviations

**NHM**Natural History Museum, London, UK.

**CUK** personal collections of P. Ustjuzhanin and V. Kovtunovich, Novosibirsk and Moscow, Russia.

**NECJU** Nature Education Centre, Jagiellonian University, Kraków, Poland.

**MNHN**Museum National d'Histoire Naturelle, Paris, France.

**TMSA**Ditsong National Museum of Natural History (formerly Transvaal Museum), Pretoria, South Africa.

**ZMHB**Museum für Naturkunde, Berlin, Germany.

**ZISP**Zoological museum of St. Petersburg, Russia.

### Sampling localities

**Figure 1.**
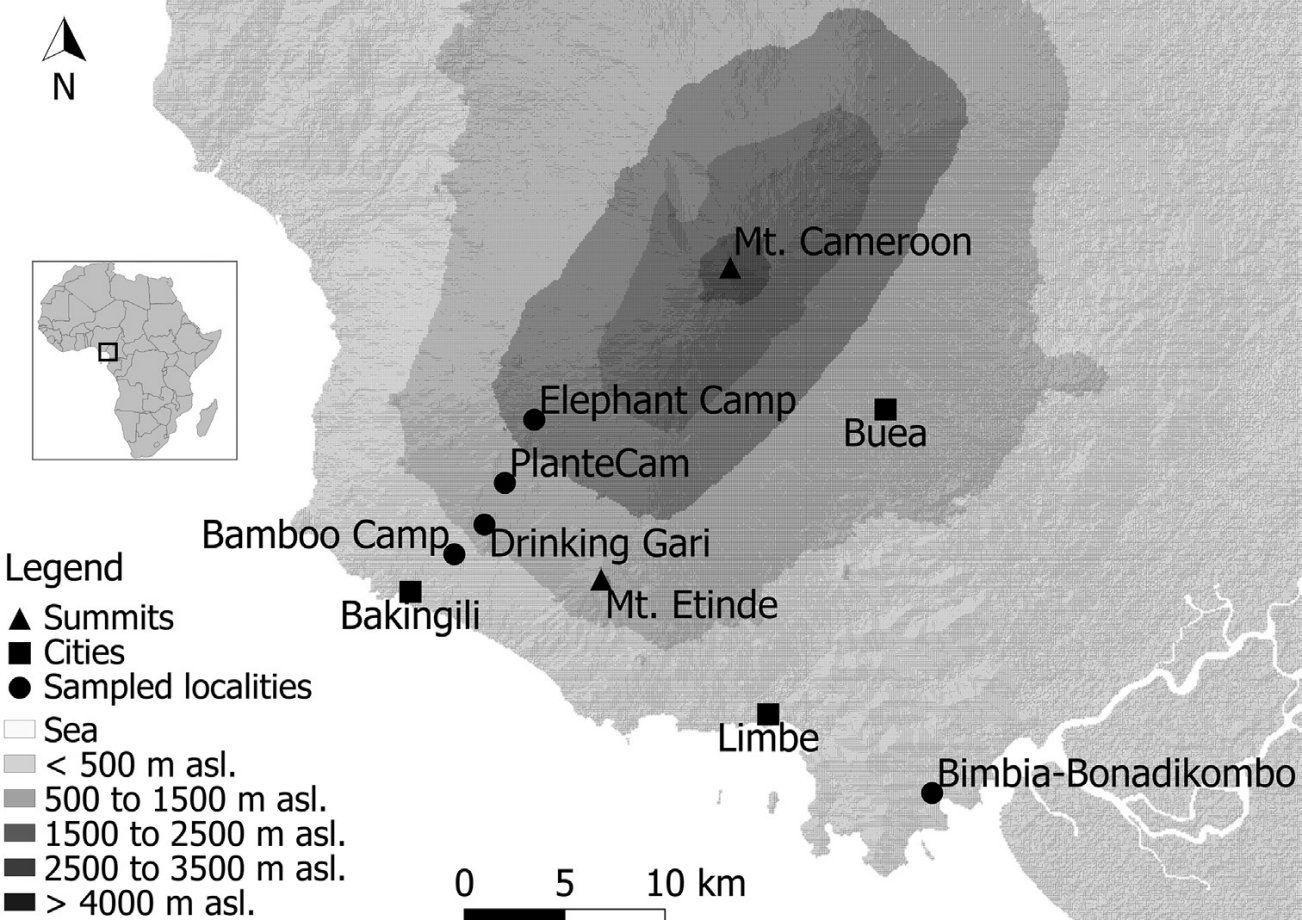
Map of the study area and localities.

All the presented material was sampled in rainforests of the Mount Cameroon National Park and in the littoral forest of the nearby Bimbia-Bonadikombo Community Forest, both lying in the Southwest Province, Cameroon (Fig. 1). This paper presents data from the first two field expeditions, when mostly the lower altitudinal zones were sampled, with the exception of mountain forests around the Elephant Camp. The two reported field trips comprised sampling in a transition from wet to dry seasons (November/December 2014 – Bamboo Camp, Drinking Gari Camp, Elephant Camp, PlanteCam), a full dry season (December 2014/January 2015 – Bimbia-Bonadikombo), and a transition from dry to wet seasons (April/May 2015 – Bamboo Camp, Drinking Gari Camp, Bimbia-Bonadikombo, PlanteCam). All the sampled localities are listed here:

Bamboo Camp – Bamboo Camp (350 m a.s.l.), Mount Cameroon (SW slope), N4.08791°, E9.05051°; a lowland forest with historical disturbances by selective logging.

Bimbia-Bonadikombo – Mexico Camp (30 m a.s.l.), Bimbia-Bonadikombo Community Forest, N3.98183°, E9.26250°; a littoral forest in the formally non-intervention part of the Community forest, but with intensive current logging (Ferenc et al. in press)

Drinking Gari – Drinking Gari Camp (650 m a.s.l.), Mount Cameroon (SW slope), N4.10144°, E9.06100°; a lowland forest with a presumably closed canopy layer.

Elephant Camp – Elephant Camp (1850 m a.s.l.), Mount Cameroon (SW slope), N4.11700°, E9.07292°; a montane forest with a highly sparse canopy layer, as a consequence of intensive natural disturbances by forest elephants.

PlanteCam – PlanteCam Camp (1100 m a.s.l.; also misspelled as “Planticamp”), Mount Cameroon (SW slope), N4.11750°, E9.07094°; an upland forest in the transition between lowland and montane zones, its canopy layer is substantially opened by natural disturbances by forest elephants.

### Sampling methods

In each of the above-listed localities, three sampling plots were established to partially cover the heterogeneity of local forest habitats. In each plot, moths were collected during two full nights (from dusk till dawn) in each sampled season. All specimens were captured at an artificial light (a single energy saving bulb: 4100 K, 5300 lm, 105 W, 5 U), powered by a portable generator. The bulb was set in the centre of two perpendicularly placed white sheets (1.5 × 1.5 × 1.8 m, the cloth type B, produced by Entosphinx, Czechia). Specimens were killed by ammonia vapours and either pinned in situ or temporarily stored in glassine envelopes, all dried by silica gel. Later, all specimens were properly mounted in the lab.

### Genitalia preparations

The preparation of genitalia slides is necessary for the identification of Alucitidae. Normally, abdomens were boiled in a 10–15% solution of potassium hydroxide until semi-transparent. After this, they were rinsed in water thoroughly for the preparation of permanent slides. On the mount, genitalia were put in a small drop of Euparal after being dehydrated in 100% ethanol. The mounts were then covered with a cover glass. In case the genitalia structures were not well sclerotised, they were stained with Chlorazol Black for greater contrast. Permanent preparations were dried for at least two weeks before being studied. Each permanent preparation received its unique code under which it is searchable in the collections where they are stored; the relevant numbers are also mentioned in captions of the genitalia figures.

## Results

### Recorded species

Altogether 15 species of Alucitidae were recorded on Mount Cameroon during the reported two field expeditions: nine of them proved to be new for science, and four others were new country records for Cameroon (marked with *), only two had been recorded previously. The morphological terminology follows Zagulajev (1986). The distribution of individual species follows the Afromoths database (De Prins and De Prins 2018).

#### * Alucita acalyptra

Taxon classificationAnimaliaLepidopteraAlucitidae

(Meyrick, 1913)

Orneodes acalyptra Meyrick, 1913: 269. Type locality: Barberton, Republic of South Africa. Holotype: male, TMSA, examined by the authors.

##### Material examined.

**Bamboo Camp**, 1 female, (CUK), 17–23.IV.2015, V. Maicher, Sz. Sáfián, Š. Janeček, R. Tropek.

##### Diagnosis.

In the mottled grey colour of the wings with contrast zigzag bands, this species is close to *A.agassizi* Ustjuzhanin & Kovtunovich, 2018. In the male genitalia, the shape of the phallus and the lanceolate uncus are similar to *A.hemicyclus* (Hering, 1917), but it differs by the blunt gnathos and the basally narrow valves.

##### Distribution.

Malawi, Republic of South Africa, Cameroon.

#### * Alucita chloracta

Taxon classificationAnimaliaLepidopteraAlucitidae

(Meyrick, 1908)

Orneodes chloracta Meyrick, 1908: 507. Type locality: Benin. Holotype: female, BMNH, examined by the authors.

##### Material examined.

**PlanteCam**, 5 ex., (CUK, NECJU), 11–18.XII.2014; **Elephant Camp**, 18 ex., 19–24.XI.2014; **Bamboo Camp**, 7 ex., (CUK, NECJU), 17–23.IV.2015; **Drinking Gari**, 1 male, (CUK), 11–23.IV.2015, V. Maicher, Sz. Sáfián, Š. Janeček, R. Tropek.

##### Diagnosis.

In the female genitalia, the shape of the bursa copulatrix and the round signum, resemble those of *A.entoprocta* (Hering, 1917), from which it differs by the wide ductus, the narrow antrum, and by the wings pattern.

##### Distribution.

Benin, Cameroon.

#### * Alucita coffeina

Taxon classificationAnimaliaLepidopteraAlucitidae

(Viette, 1958)

Orneodes coffeina Viette, 1958: 457. Type locality: Oubangui-Chari, Boukoko, [Central African Republic]. Holotype: female, MNHN, examined by the authors.

##### Material examined.

**PlanteCam**, 1 male, (CUK), 11–18.XII.2014, V. Maicher, Sz. Sáfián, Š. Janeček, R. Tropek.

##### Diagnosis.

The bright orange wings with dark, almost black bases and the large size (about 20 mm), are unique for this species. In the male genitalia, the blunt apex of the uncus and the narrow anellus arms, are close to *A.crococyma* (Meyrick, 1937), from which it differs by the short triangular valves, the narrow gnathos and the needle-like clusters of cornuti in the distal part of the phallus.

##### Distribution.

Central African Republic, Cameroon.

#### Alucita megaphimus

Taxon classificationAnimaliaLepidopteraAlucitidae

(Hering, 1917)

Orneodes megaphimus Hering, 1917: 191. Type locality: Cameroon. Holotype: male, ZMHB, examined by the authors.

##### Material examined.

**Bamboo Camp**, 6 ex., (CUK, NECJU), 17–23.IV.2015; **PlanteCam**, 5 ex., (CUK, NECJU), 09–14.IV.2015; **Drinking Gari**, 3 ex., (CUK, NECJU), 11–23.IV.2015, V. Maicher, Sz. Sáfián, Š. Janeček, R. Tropek.

##### Diagnosis.

In the mottled colour of the wings with zigzag white bands, the species is similar to *A.seychellensis* (Fletcher, 1910), but differs by the larger size and the elongated fore wings. In the male genitalia, it differs from *A.seychellensis* by the notch at the apex of the uncus, more narrow valves, and robust serrated saccular processes.

##### Distribution.

Cameroon.

#### * Alucita spicifera

Taxon classificationAnimaliaLepidopteraAlucitidae

(Meyrick, 1911)

Orneodes spicifera Meyrick, 1911: 221. Type locality: Pretoria, Republic of South Africa. Holotype: male, TMSA, examined by the authors.

##### Material examined.

**PlanteCam**, 1 male, (NECJU), 11–18.XII.2014, 4 males, (CUK, NECJU), 09–14.IV.2015; **Elephant Camp**, 7 males, (CUK, NECJU), 19–24.XI.2014, V. Maicher, Sz. Sáfián, Š. Janeček, R. Tropek.

##### Diagnosis.

In the male genitalia, the species is close to the Palaearctic species of *Alucita*. In particular, in its crown-shaped uncus, narrow membranous valves and long anellus arms, the species is similar to *A.cinnerethella* (Amsel, 1935), known from Iran, Turkey and Israel. However, it is distinctive in the shorter phallus, in the widened, almost round apex of the gnathos, and also in the wing colour.

##### Distribution.

Republic of South Africa, Malawi, Tanzania, Cameroon.

#### Alucita zinovievi

Taxon classificationAnimaliaLepidopteraAlucitidae

Kovtunovich & Ustjuzhanin, 2016

Alucita zinovievi Kovtunovich & Ustjuzhanin, 2016: 299. Type locality: PlanteCam, Moutn Cameroon, Cameroon. Type: male, ZISP, examined by the authors.

##### Material examined.

PlanteCam, 1100 m a.s.l., Mount Cameroon (SW slope), N4.1175000°, E9.0709440°, 11–18.XII.2014. V. Maicher, Sz. Sáfián, Š. Janeček, R. Tropek.

##### Diagnosis.

In the male genitalia, the shape of the uncus, gnathos, and phallus, this species is similar to *A.aarviki* Ustjuzhanin & Kovtunovich, 2016. These species differ from each other by the wider valves, long anellus arms and cornuti. The new species is also distinctive in the wing colour: in *A.aarviki* the wings are yellow with white transverse bands, while in *A.zinovievi*, the wings are white with wide dark brown bands (Kovtunovich and Ustjuzhanin 2016).

##### Distribution.

Cameroon.

#### Alucita longipenis

Taxon classificationAnimaliaLepidopteraAlucitidae

Ustjuzhanin & Kovtunovich sp. n.

http://zoobank.org/0C5D41E9-38EE-4A7C-BB72-56855C9066D7

##### Type material.

**Holotype**, male, (NECJU 201801) **CAMEROON**, **Elephant Camp**, 1850 m a.s.l., Mount Cameroon (SW slope), N4.11700°, E9.07292°, 19–24.XI.2014, V. Maicher, Sz. Sáfián, Š. Janeček, R. Tropek. **Paratypes**: 4 males, 2 females (NECJU, CUK) same data as holotype; 1 male (CUK), **PlanteCam**, 09–14.IV.2015, V. Maicher, Sz. Sáfián, Š. Janeček, R. Tropek.

**Figures 2–4.**
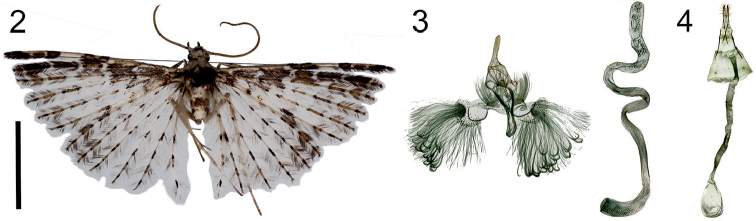
*Alucitalongipenis* Ustjuzhanin & Kovtunovich, sp. n. **2** Adult male, Holotype, NECJU**3** Male genitalia, Holotype, NECJU, preparation slide no. 201801 **4** Female genitalia, Paratype, NECJU, preparation slide no. 201810. Scale bar: 5 mm.

##### Diagnosis.

In the wings’ colour and the male genital structure, the new species is unique, with no analogues among known African Alucitidae. The phallus length, disproportionate in relation to the entire genital structure, distinguishes this species from all the known species of this family.

##### External characters.

Head with white spiky hairs, thorax, and tegula white with portions of brown strokes. Labial palpus thin, straight, 2 × longer than longitudinal eye diameter; brown on the outside, white on the inside. Third segment thin, narrow, tapered to apex. Antenna white, scape laterally thickened. Wingspan 18–23 mm, of holotype 22 mm. Wings white, with patches of brown strokes and spots. Small, dark brown rectangular spot in basal part of first lobe of fore wing. Larger, pale brown elongated spot with triangle cut in middle part of lobe. Alternating white, pale brown, and dark brown patches in the distal part of the first lobe. Apical part darkened with black scales. Dark brown elongated patches separated by narrow white bands on second lobe. Alternating brown and white elongated portions of scales on other four lobes of fore wing. Lobes of hind wing white, with patches of elongated brown strokes and spots. Fringe on wing pale, between first and second lobe of fore wing fringe with portions of dark brown scales. Abdomen white, with small spots of dark brown scales. Hind leg pale yellow.

##### Male genitalia.

Uncus long, equally wide along entire length. Gnathos longer than uncus, sharply thickened at end. Gnathos arms narrow, tapered to apices. Valva reduced, poorly expressed. Anellus arms long, narrow, slightly widened at apices. Saccus very long, elongated, smoothly curved in distal part. Phallus extremely long: 5 × longer than the entire genital structure, sharp arched bands in middle part, without cornuti.

##### Female genitalia.

Papilla analis narrow, elongated. Posterior apophyses straight, thin, long. Anterior apophyses equal in length to posterior apophyses, but slightly thicker and undulated. Antrum narrow, tubulate, length almost equal to posterior apophyses. Ductus corrugated, thin, very long: 4 × longer than antrum. Bursa copulatrix small, oval, barely exceeds length of antrum, without signa.

##### Distribution.

Cameroon.

##### Flight period.

April, November.

##### Etymology.

The species is named after the morphological peculiarity of the phallus; the species name is a noun in apposition.

#### Alucita lidiya

Taxon classificationAnimaliaLepidopteraAlucitidae

Ustjuzhanin & Kovtunovich sp. n.

http://zoobank.org/0149F089-D5B7-47BD-BF49-B8A5FAF14FC1

##### Type material.

**Holotype**, male, (NECJU 201802) **CAMEROON**, **Bamboo Camp**, 350 m a.s.l., Mount Cameroon (SW slope), N4.08791°, E9.05051°, 17–23.IV.2015. V. Maicher, Sz. Sáfián, Š. Janeček, R. Tropek. **Paratypes**: 1 male (CUK), **Drinking Gari**, 11–23.IV.2015; 2 males (NECJU, CUK), **PlanteCam**, 09–14.IV.2015, V. Maicher, Sz. Sáfián, Š. Janeček, R. Tropek.

**Figures 5–6.**
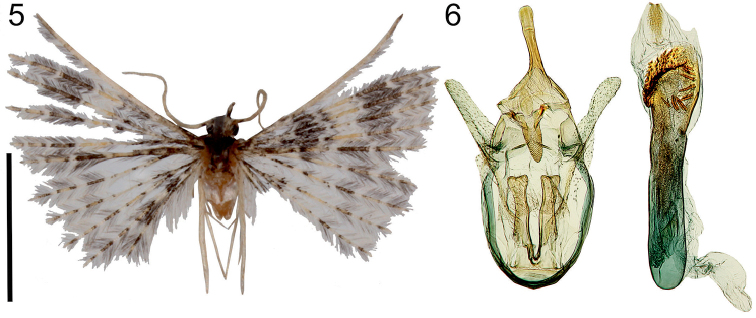
*Alucitalidiya* Ustjuzhanin & Kovtunovich, sp. n. **5** Adult male, Holotype, NECJU**6** Male genitalia, Holotype, NECJU, preparation slide no. 201802. Scale bar: 5 mm.

##### Diagnosis.

The mottled pattern of the wings and position of the bands of the new species is similar to *A.chloracta* (Meyrick, 1908). These species differ from each other by the pale yellow median band in *A.lidiya*, while in *A.chloracta* the band is brown. *A.lidiya* has paler hind wings, without saturated dark grey portions of fringe, as in *A.chloracta*. Male genitalia of the new species also have some similarity to *A.chloracta*, but there are significant differences in the shape of the narrow valva, the wide gnathos and the wide anellus arms of *A.lidiya*; while in *A.chloracta* the valva is significantly wider and rounded distally, the gnathos is thin, and the anellus arms are narrow.

##### External characters.

Head, thorax and tegula with dark grey clinging hairs. Labial palpus dark grey, short, slightly longer than longitudinal eye diameter. Third segment thin, short, white, tapered to apex. Antenna pale grey. Wingspan 14–15 mm, of holotype 14 mm. Wing greyish brown. Base of fore and hind wing coloured with dark brown scales. Wide pale yellow band in median part, wide dark brown band in distal part of wing. Fringe on wing with alternating portions of pale and brown hairs. Hind leg pale yellow.

##### Male genitalia.

Uncus quite long, narrow, slightly widened distally. Gnathos short, wide, shorter than uncus. Gnathos arms thin, undulate, widened to apices. Valva narrow, quite long, poorly sclerotised. Anellus arms long, wide, equal to valva in length. Phallus short, almost straight, group of various cornuti in the distal part: fine needle-shaped and large, with serrated edges.

##### Distribution.

Cameroon.

##### Flight period.

April.

##### Etymology.

The species name is a noun in apposition in honour of Lidiya Bezverkhova.

#### Alucita ludmila

Taxon classificationAnimaliaLepidopteraAlucitidae

Ustjuzhanin & Kovtunovich sp. n.

http://zoobank.org/6B6DA5D1-494E-47E5-A362-9DB51258D5EC

##### Type material.

**Holotype**, male, (NECJU 201803) **CAMEROON**, **Bamboo Camp** (350 m a.s.l.), Mount Cameroon (SW slope), N4.08791°, E9.05051°, 17–23.IV.2015. V. Maicher, Sz. Sáfián, Š. Janeček, R. Tropek. **Paratypes**: 1 male (NECJU), **Bamboo Camp**, 12–20.XII.2014; 1 male (CUK), **PlanteCam**, 11–18.XII.2014; 1 male (CUK), **Drinking Gari**, 11–23.IV.2015, V. Maicher, Sz. Sáfián, Š. Janeček, R. Tropek; 1 male (NHM), **NIGERIA**, **Forest Sapobc** (?), 17.IV.1976, M.A. Comes; 1 male (NHM), **NIGERIA**, **Gambari Forest**, Oyo State, 2.X.1976, M.A. Comes; 1 male (NHM), **NIGERIA**, **Gambari Forest**, Oyo State, 8.X.1977, J. Riley; 1 female (NHM), **NIGERIA**, **Port Harcourt**, Rivers, 2.VI.1958, B.J. MacNutly; 1 male (NHM), **NIGERIA**, Crin, 30.VII.1976, M.A. Comes; 1 female (CUK), **GHANA**, **Bunso Arboretum**, Eastern Region, 16–18.XI.2011, Sz. Sáfián, F. Pühringer.

**Figures 7–9.**
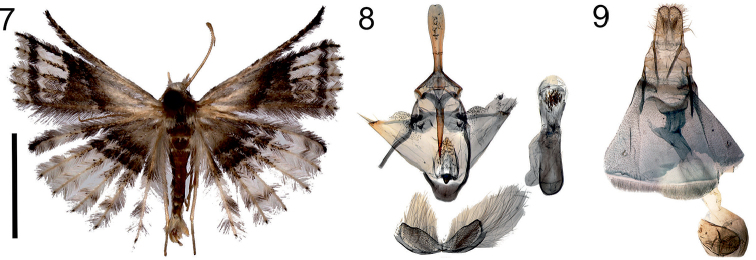
*Alucitaludmila* Ustjuzhanin & Kovtunovich, sp. n. **7** Adult male, Holotype, NECJU**8** Male genitalia, Holotype, NECJU, preparation slide no. 201803 **9** Female genitalia, Paratype, CUK, preparation slide no. 451. Scale bar: 5 mm.

##### Diagnosis.

In the wing colour, this new species is unique among the African Alucitidae. In the male genitalia structure, the wing-shaped valva and shape of the phallus are similar to *A.aarviki* Ustjuzhanin & Kovtunovich, 2016, but it differs from that by the shape of the uncus, the tapered apices of the valva, the narrow arms of the anellus and the cluster of needle-shaped cornuti in the distal part of the phallus.

##### External characters.

Head, thorax, and tegula with greyish white clinging hairs. Labial palpus greyish white, almost 2 × longer than longitudinal eye diameter. Third segment thin, short, white, tapered to apex. Antenna pale brown. Wingspan 16–23mm, of holotype 17 mm. Wing colour greyish white. Base of wing pale grey, wide brownish grey band in median part. Well pronounced pale elongated patch, framed by dark brown band in distal part. Portions of dark brown hairs in distal part of hind wing. Fringe on wing with alternating portions of pale and brown hairs. Hind leg pale yellow.

##### Male genitalia.

Uncus long, paddle-like, with even edge of apex. Gnathos longer than uncus, narrow, tapered to apex. Gnathos arms short, wide. Valva wing-like, triangle, long needle-shaped bristles in apical part. Anellus arms short, narrow. Saccus short, with even outer edge. Phallus quite short, almost straight, cluster of small needle-shaped cornuti in distal part.

##### Female genitalia.

Papilla analis wide, elongated. Posterior apophyses straight, short, slightly longer than the papilla analis. Anterior apophyses thicker than posterior ones, equal to them in length. Ostium wide, cupped. Antrum tubulate, short, wide, sclerotised, almost equal to length of posterior apophyses. Ductus wide at confluence to antrum, twice as wide as antrum, ductus seminalis short. Bursa copulatrix round, without signa.

##### Distribution.

Cameroon, Nigeria, Ghana.

##### Flight period.

From April to December.

##### Etymology.

The species name is a noun in apposition in honour of the first author’s wife, Ludmila Ustjuzhanina.

#### Alucita escobari

Taxon classificationAnimaliaLepidopteraAlucitidae

Ustjuzhanin & Kovtunovich sp. n.

http://zoobank.org/D1CF050D-CB13-4283-87C2-5DE4D082E730

##### Type material.

**Holotype**, male, (NECJU 201804) **CAMEROON**, PlanteCam, 1100 m a.s.l., Mount Cameroon (SW slope), N4.11750°, E9.07094°, 11–18.XII. 2014. V. Maicher, Sz. Sáfián, Š. Janeček, R. Tropek. **Paratypes**: 1 female (CUK), same data as holotype; 2 males (NECJU, CUK), **Bamboo Camp**, 12–20.XII.2014, V. Maicher, Sz. Sáfián, Š. Janeček, R. Tropek.

**Figures 10–12.**
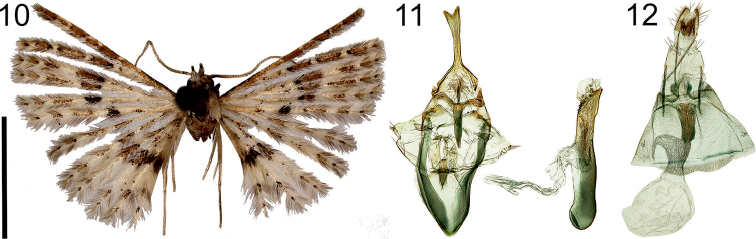
*Alucitaescobari* Ustjuzhanin & Kovtunovich, sp. n. **10** Adult male, Holotype, NECJU**11** Male genitalia, Holotype, NECJU, preparation slide no. 201804 **12** Female genitalia, Paratype, CUK, preparation slide no. 452. Scale bar: 5 mm.

##### Diagnosis.

The mottled colour and the median arched band on the wings of the new species is similar to *A.mischenini*. The new species is distinguished by the brown spot of scales on the sixth lobe of the fore wing, declining from the correct arc and shifting towards the basal part of the lobe. In the male genital structure, the uncus with the apical triangle cut and the phallus obliquely cut at apex of the new species are similar to *A.balioxantha* Meyrick, 1921. These species differ from each other by the valva in the shape of a wide-triangle valva, the elongated saccus, and the tapered gnathos in *A.escobari*.

##### External characters.

Head with white spiky hairs, thorax, and tegula white. Labial palpus quite wide, short, 1.5 × longer than longitudinal eye diameter, slightly bent upwards, painted with brown scales inside and outside. Third segment short, white on apex. Antenna yellowish brown, scape thickened. Wingspan 14–16 mm, of holotype 15.5 mm. Wing yellowish brown. Transverse brown arched band well expressed in median part of both wings. On sixth lobe of fore wing, patch of scales declining from correct arc and shifting towards basal part of lobe. Wing base pale, interspersed with small brown scales. Alternating brown and white elongated portions of scales in distal part of fore wing. Fringe on wing yellow with rare portions of brown hairs. Hind leg yellow.

##### Male genitalia.

Uncus long, widened distally, triangle cut apically. Gnathos slightly shorter than uncus, thick, tapered to apex. Gnathos arms short, thick, smoothly bent inwards. Valva short, wide-triangle, apically with bunch of thin needle-shaped bristles. Anellus arms wide, slightly shorter than gnathos, slightly bent inwards, narrowed apically. Saccus elongated, narrow-triangle, sharp outer edge. Phallus almost straight, apex obliquely cut, small needle-shaped cornuti medially and distally.

##### Female genitalia.

Papilla analis narrow, triangle. Posterior apophyses straight, long, thin. Anterior apophyses thicker than posterior, equal to them in length. Antrum tubulate, sclerotised, length equal to posterior apophyses. Ductus wide at confluence with antrum, twice as wide as antrum, ductus seminalis short. Bursa copulatrix round, without signa.

##### Distribution.

Cameroon.

##### Flight period.

December.

##### Etymology.

The authors name the species in recognition of Francis Luma Ewome, locally well known as ‘Escobar’, a very well trained guide on Mount Cameroon. Over the years, he became instrumental in organising and implementing the field expeditions, and also became a good friend to the last three authors of this paper. It could be stated that the research would have been extremely difficult without the selfless help of Escobar.

#### Alucita mischenini

Taxon classificationAnimaliaLepidopteraAlucitidae

Ustjuzhanin & Kovtunovich sp. n.

http://zoobank.org/ACC44B4D-76B6-4364-86CB-90BA6665C3B6

##### Type material.

**Holotype**, male, (NECJU 201805) **CAMEROON**, **Bimbia-Bonadikombo**, 30 m a.s.l., Mexico Camp, Bimbia-Bonadikombo Community Forest, N3.98183°, E9.26250°, 07–12.V.2015, V. Maicher, Sz. Sáfián, Š. Janeček, R. Tropek. **Paratypes**: 2 males (NECJU, CUK), **PlanteCam**, 11–18.XII.2014, V. Maicher, Sz. Sáfián, Š. Janeček, R. Tropek.

**Figures 13–14.**
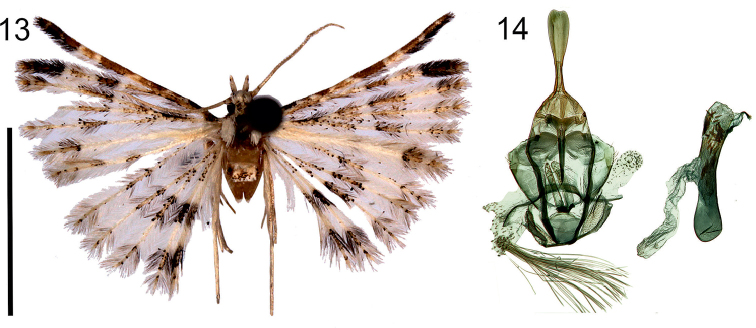
*Alucitamischenini* Ustjuzhanin & Kovtunovich, sp. n. **13** Adult male, Holotype, NECJU**14** Male genitalia, Holotype, NECJU, preparation slide no. 201805. Scale bar: 5 mm.

##### Diagnosis.

The mottled colour and the transverse arched band on the wings of *A.mischenini* are similar to *A.escobari*. These species differ from each other by the brown spot of scales on the sixth lobe of the fore wing, declining from the arc to the distal part of the lobe. In the male genital structure, the shape of the valva and the phallus is also similar to *A.escobari*. These species differ from each other by the shape of the uncus, the longer gnathos, the saccus wide in the base, and narrower, longer anellus arms in *A.mischenini*.

##### External characters.

Head, thorax and tegula with white clinging scales. Labial palpus short, 1.5 × longer than longitudinal eye diameter, directed forward, white on inside, coloured with small brown scales on outside. Third segment short, middle part framed in narrow brown band, apex tapered. Antenna yellow. Wingspan 12–15 mm, of holotype 12 mm. Wing mottled, median transverse band of brown elongated spots of scales developed on first five lobes of fore wing. Similar spot on sixth lobe, declining from band and shifted to distal part of lobe. Median transverse band on hind wing forms correct arc. Small dark brown scales in basal part of fore and hind wing. Alternating portions of brown and yellow scales in distal part. Fringe on wing yellow, with alternating rare portions of brown hairs. Hind leg white.

##### Male genitalia.

Uncus long, basally narrow, distally wide, with poorly expressed cut at apex. Gnathos significantly longer than uncus, narrow, tapered to apex. Gnathos arms short, thick, smoothly bent inwards. Median process between gnathos arms well developed. Valva wing-like, short, wide. Anellus arms long, slightly shorter and noticeably wider than gnathos. Saccus short, basally wide, small oval cut on outer edge. Phallus short, slightly bent in middle, distally with small needle-shaped cornuti.

##### Distribution.

Cameroon.

##### Flight period.

May, December.

##### Etymology.

The species is named after the Novosibirsk biologist and naturalist Sergei Ivanovich Mischenin.

#### Alucita fokami

Taxon classificationAnimaliaLepidopteraAlucitidae

Ustjuzhanin & Kovtunovich sp. n.

http://zoobank.org/A61D9EE8-F9A8-4A57-97AA-F21CAC659328

##### Type material.

**Holotype**, male, (NECJU 201806) **CAMEROON**, **Bamboo Camp**, 350 m a.s.l., Mount Cameroon (SW slope), N4.08791°, E9.05051°, 17–23.IV.2015, V. Maicher, Sz. Sáfián, Š. Janeček, R. Tropek. **Paratypes**: 1 female (NECJU), same data as holotype; 1 male (CUK), **PlanteCam**, 11–18.XII. 2014; 2 males, 1 female (NECJU, CUK), **Drinking Gari**, 11–23.IV.2015, V. Maicher, Sz. Sáfián, Š. Janeček, R. Tropek.

**Figures 15–17.**
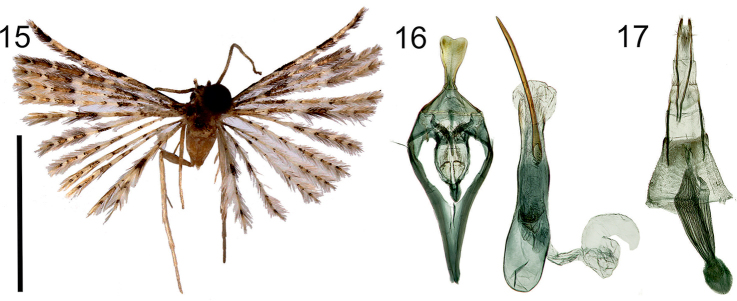
*Alucitafokami* Ustjuzhanin & Kovtunovich, sp. n. **15** Adult male, Holotype, NECJU**16** Male genitalia, Holotype, NECJU, preparation slide no. 201806 **17** Female genitalia, Paratype, NECJU, preparation slide no. 201811. Scale bar: 5 mm.

##### Diagnosis.

The mottled colour of the wings and the median arched band on the fore wing in *A.fokami*. is similar to *A.escobari*. *A.fokami* is characterised by the absence of such a band on the hind wings and by the base of the wings darkened with brown scales. In the male genitalia, the absence of the valva in the new species is similar to *A.janeceki*. These species differ from each other by the long needle-shaped cornutus, narrow elongated saccus, and the shape of the uncus in *A.fokami*.

##### External characters.

Head with pale yellow spiky hairs, thorax and tegula pale brown. Labial palpus yellow-brown, 1.5 × longer than eye diameter. Antenna yellow. Wingspan 12–13 mm, of holotype 12 mm. Wing yellowish brown with three white transverse bands. Wing base darkened with brown scales. Fringe on wing yellow. Hind leg white.

##### Male genitalia.

Uncus basally narrow, distally wide, apex with small oval cut. Gnathos short, 3 × smaller than uncus, narrow, slightly tapered to apex. Gnathos arms thick, long, undulate, apically tapered. Median process between gnathos arms well developed. No valva. Anellus arms very short, in shape of wide lobes, equal to gnathos in length. Saccus narrow, elongated, exceeds length of uncus with tegumen. Phallus short, almost straight, distally with long needle-shaped cornutus exceeding total length of phallus.

##### Female genitalia.

Papilla analis narrow, elongated. Posterior apophyses very long, slightly undulated. Anterior apophyses straight, long, slightly shorter than posterior apophyses. Antrum narrow, short. Ductus long, corrugated, with well-expressed longitudinal cords. Ductus narrow at confluence to bursa copulatrix. Ductus seminalis short, wide. Bursa copulatrix oval, small, equal to papilla analis, without signa.

##### Distribution.

Cameroon.

##### Flight period.

April, December.

##### Etymology.

The authors name this species in recognition of Dr. Eric Bertrand Fokam, the current head of the Department of Zoology and Animal Physiology, University of Buea. Eric is a renowned ecologist and a keen field scientist. He has not only been an active collaborator during the field research on Mount Cameroon, but has also brought up a new generation of young Cameroonians to continue the scientific work on insects and other animal groups in Cameroon.

#### Alucita janeceki

Taxon classificationAnimaliaLepidopteraAlucitidae

Ustjuzhanin & Kovtunovich sp. n.

http://zoobank.org/2753F767-627A-4882-B138-E8DB95800AE7

##### Type material.

**Holotype**, male, (NECJU 201807) **CAMEROON**, **Bamboo Camp**, 350 m a.s.l., Mount Cameroon (SW slope), N4.08791°, E9.05051°, 17–23.IV.2015, V. Maicher, Sz. Sáfián, Š. Janeček, R. Tropek. **Paratypes**: 1 male, 1 female (NECJU, CUK), same data as holotype; 1 male (CUK), **Drinking Gari**, 11–23.IV.2015, V. Maicher, Sz. Sáfián, Š. Janeček, R. Tropek.

**Figures 18–20.**
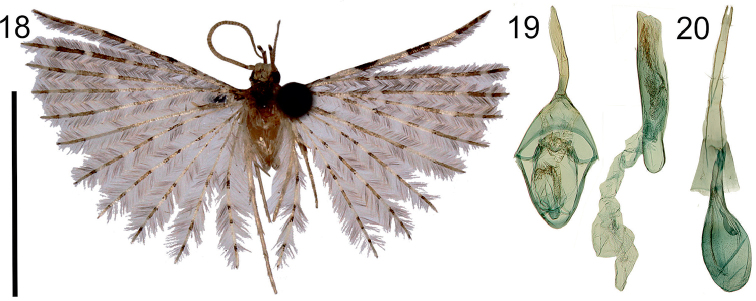
*Alucitajaneceki* Ustjuzhanin & Kovtunovich, sp. n. **18** Adult male, Holotype, NECJU**19** Male genitalia, Holotype, NECJU, preparation slide no. 201807 **20** Female genitalia, Paratype, CUK, preparation slide no. 453. Scale bar: 5 mm.

##### Diagnosis.

The wings colour and the absence of the valva in the male genitalia of the new species is similar to *A.fokami*. In the male genitalia, *A.janeceki* is characterised by the oval saccus, the long uncus slightly tapered to apex, the long and wide phallus without a large needle-shaped cornutus. In the female genitalia, the new species is characterised by the oval bursa copulatrix with the ductus seminalis inside, and by the very long posterior apophyses.

##### External characters.

Head, thorax and tegula yellowish white, interspersed with fine brown scales. Labial palpus white, 2 × longer than eye diameter, slightly bent upwards. Third segment thin, tapered to apex. Antenna pale brown. Wingspan 10–12 mm, of holotype 12 mm. Wing greyish white. Wing base darkened with brown scales. Well expressed elongated brown strokes separated by yellowish portions on first lobe. Apical part of lobe brown, sharp. Second and other lobes repeat the pattern of first but with less contrast. Fringe on wing with alternating pale and brown hairs. Hind leg pale yellow.

##### Male genitalia.

Uncus very long, distally wide, apically slightly tapered. Gnathos not expressed. Valva reduced. Anellus arms straight, wide, twice as short as uncus. Saccus oval. Phallus long and wide, 1.5 × longer than uncus, almost straight, medially and distally with clusters of small spiny cornuti.

##### Female genitalia.

Papilla analis narrow, elongated. Posterior apophyses very long, thin. Anterior apophyses straight, very long, equal to posterior apophyses. Antrum short, V-shaped. Ductus wide, corrugated, with longitudinal cords and clusters of small signa. Ductus seminalis inside bursa copulatrix, long, distally widened. Bursa copulatrix big, oval, with impregnation of small spiny signa.

##### Distribution.

Cameroon.

##### Flight period.

April.

##### Etymology.

The species is named after Dr. Štěpán Janeček, an experienced botanist who accompanied all our field expeditions and crucially helped us with many things, including the collection of a substantial part of the presented specimens.

#### Alucita besongi

Taxon classificationAnimaliaLepidopteraAlucitidae

Ustjuzhanin & Kovtunovich sp. n.

http://zoobank.org/2753F767-627A-4882-B138-E8DB95800AE7

##### Type material.

**Holotype**, male, (NECJU 201808) **CAMEROON**, **Bamboo Camp**, 350 m a.s.l., Mount Cameroon (SW slope), N4.08791°, E9.05051°, 17–23.IV.2015, V. Maicher, Sz. Sáfián, Š. Janeček, R. Tropek. **Paratypes**: 2 females (NECJU, CUK), same data as holotype.

**Figures 21–23.**
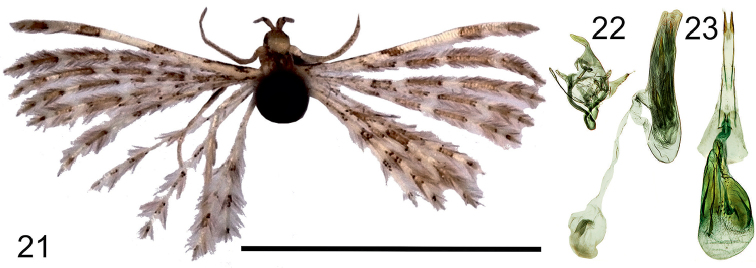
*Alucitabesongi* Ustjuzhanin & Kovtunovich, sp. n. **21** Adult male, Holotype, NECJU. **22** Male genitalia, Holotype, NECJU, preparation slide no. 201808. **23** Female genitalia, Paratype, NECJU, preparation slide no. 201812. Scale bar: 5 mm.

##### Diagnosis.

The yellowish brown wings and the small size of the new species are similar to *A.certifica* Meyrick, 1909. These species differ from each other by the absence of the wide pale-brown band in the median portion of the fore wing in *A.besongi*. In the male genitalia, the shape of the uncus and the gnathos of the new species have some similarities to those of *A.acalyptra* Meyrick, 1913; these species differ from each other by the elongated saccus, the peculiar shape of the valva and the phallus with large needle-shaped cornuti in *A.besongi*.

##### External characters.

Head, thorax, and tegula with white clinging scales. Labial palpus white, interspersed with small brown scales, 2 × longer than longitudinal eye diameter, bent upwards. Third segment thin, framed with narrow brown band at base. Antenna yellow. Wingspan 8–10 mm, of holotype 9 mm. Wing yellowish brown. Wing base interspersed with small brown scales. White longitudinal strokes separated by pale brown portions well expressed on first lobe. Poorly noticeable series of white bands from wing base to apical area. Fringe on wing yellowish brown. Hind leg pale yellow.

##### Male genitalia.

Uncus long, distally wide, apically slightly tapered. Gnathos narrow, long, tapered to apex. Valva basally wide, then smoothly narrowing. Anellus arms straight, long. Saccus elongated, oval. Phallus thick, long, almost twice as long as entire genital structure, with cluster of large spiny cornuti.

##### Female genitalia.

Papilla analis narrow, elongated. Posterior apophyses long, thin. Anterior apophyses straight, equal to posterior apophyses. Antrum short, V-shaped. Ductus short, corrugated, with longitudinal cords, smoothly turning into bursa copulatrix. Ductus seminalis inside bursa copulatrix. Bursa big, oval, with long ribbon-like signa and impregnation of small spiny signa.

##### Distribution.

Cameroon.

##### Flight period.

April.

##### Etymology.

The species is named after Simon B. Besong, the current main conservator of the Mount Cameroon National Park, who helped our research by various means of support.

#### Alucita olga

Taxon classificationAnimaliaLepidopteraAlucitidae

Ustjuzhanin & Kovtunovich sp. n.

http://zoobank.org/461C9B8A-5BF5-46B9-9A1A-CB04F656358A

##### Type material.

**Holotype**, male, (NECJU 201809) **CAMEROON**, **Bamboo Camp**, 350 m a.s.l., Mount Cameroon (SW slope), N4.08791°, E9.05051°, 17.-23.IV.2015. V. Maicher, Sz. Sáfián, Š. Janeček, R. Tropek. **Paratypes**: 1 male, 2 females (NECJU, CUK), same data as holotype; 1 male (CUK), **PlanteCam**, 09–14.IV.2015, V. Maicher, Sz. Sáfián, Š. Janeček, R. Tropek.

**Figures 24–26.**
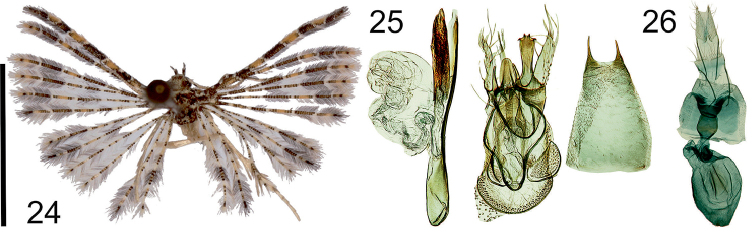
*Alucitaolga* Ustjuzhanin & Kovtunovich, sp. n. **24** Adult male, Holotype, NECJU**25** Male genitalia, Holotype, NECJU, preparation slide no. 201809 **26** Female genitalia, Paratype, NECJU, preparation slide no. 201813. Scale bar: 5 mm.

##### Diagnosis.

The mottled yellowish brown wings of the new species have some similarity to *A.lidiya*, but these species differ from each other by the narrow medial band and the dark-brown spots in the apical parts of the lobes in *A.olga*. In the male genitalia, the shapes of the uncus, saccus and phallus of *A.olga* are similar to *A.spicifera* Meyrick, 1911. These species differ from each other by the wide, oval gnathos and the short valva not widened distally, in *A.olga*, and by the gnathos and valva widened at the apices in *A.spicifera*.

##### External characters.

Head, thorax and tegula with brown-yellow clinging scales. Labial palpus spotty, with alternating white and black scales, 2 × longer than longitudinal eye diameter, bent upwards. Third segment long, thin, tapered to apex. Antenna yellow, interspersed with dark brown scales. Wingspan 10–11 mm, of holotype 11 mm. Wing mottled, yellowish brown. Wing base coloured with dark brown scales. First lobe of fore wing with well-expressed orange elongated spots, alternating with dark brown elongated spots separated by white bands. Alternation of orange and dark brown spots on other lobes of both wings. Apical area of all lobes ends with dark brown spot. Fringe on wing yellowish brown, with alternating portions of pale and brown hairs. Hind leg pale yellow.

##### Male genitalia.

Uncus medially narrow, distally widened, apex with four claw-like processes forming a kind of rake. Gnathos wide, oval. Gnathos arms straight, narrow, apically tapered. Valva quite wide, short, membranous, poorly sclerotised. Anellus arms long, wide. Saccus slightly elongated, oval. Phallus narrow, elongated, longer than entire genital structure, with one well-expressed narrow long cornutus and cluster of small needle-shaped cornuti distally.

##### Female genitalia.

Papilla analis elongated, narrow triangle. Posterior apophyses thin, straight. Anterior apophyses thin, slightly longer than posterior apophyses. Antrum wide, funnel-shaped, sclerotised. Ductus wide, short, narrow at confluence to bursa copulatrix. Ductus seminalis short, wide. Bursa copulatrix big, oval, with two narrow longitudinal ribbon-like signa, impregnation of many small spines inside the whole bursa.

##### Distribution.

Cameroon.

##### Flight period.

April.

##### Etymology.

The species name is a noun in apposition in honour of Olga Birichevskaya.

## Discussion

Our report, although covering just the first part of the sampled material, has revealed that the Mount Cameroon area is the richest known locality for Alucitidae in the whole Afrotropical Region. To the best of our knowledge, maximally only a few species are known from elsewhere in the region; they are met extremely rarely and very locally in other biogeographic areas as well. Partly, this is an artefact of incomplete sampling and relatively less attention to the group during many biodiversity surveys. On the other hand, Alucitidae were specifically focused by various lepidopterists in many places in the Afrotropics in the recent years, never resulting in such rich local biodiversity. By its 15 reported species, Mount Cameroon outnumbers all the other localities in biodiversity of many-plumed moths. The Mount Cameroon area is known to host an exceptional diversity of some other groups of organisms, including Lepidoptera (Ballesteros-Mejia et al. 2013, Maicher et al. 2016, Ferenc et al. in press). Its tremendous biodiversity is considered to be the result of its location on the border between the West African Forests and the Congolese Basin, combining species pools of both these species-rich regions, together with its own endemics (Myers et al. 2000, Maicher et al. 2016). Moreover, its complete altitudinal forest gradient from seashore to timberline comprises a few steep gradients of environmental conditions known to support high species richness.

Our results have also highlighted the poor knowledge of Lepidoptera of the studied region, despite its high importance for biodiversity and its conservation. This paper increased the known Cameroonian fauna of Alucitidae from three (De Prins and De Prins 2018) to 16 species. The nine new species of *Alucita* described in this study can be supplemented by numerous recent descriptions of new species from various lepidopteran groups (e.g., Przybyłowicz 2013, Yakovlev and Sáfián 2016, Sáfián and Tropek 2016). Moreover, our lack of knowledge is further evidenced by the faunistic importance of some of our findings. Three species reported as new for the country had their nearest known localities thousands of kilometers away, in different biogeographic regions. A comparable pattern has already been reported in Erebidae by Maicher et al. (2016). It is highly unlikely that Mount Cameroon would be a refugium for so many species of Lepidoptera occurring in different Afrotropical areas. We thus rather hypothesise that at least some of the mentioned species have a more continuous distribution area, but are just insufficiently explored.

## Supplementary Material

XML Treatment for Alucita acalyptra

XML Treatment for Alucita chloracta

XML Treatment for Alucita coffeina

XML Treatment for Alucita megaphimus

XML Treatment for Alucita spicifera

XML Treatment for Alucita zinovievi

XML Treatment for Alucita longipenis

XML Treatment for Alucita lidiya

XML Treatment for Alucita ludmila

XML Treatment for Alucita escobari

XML Treatment for Alucita mischenini

XML Treatment for Alucita fokami

XML Treatment for Alucita janeceki

XML Treatment for Alucita besongi

XML Treatment for Alucita olga

## Supplementary materials

Supplementary material 1 Table of localities Data type: occurence This is an open access article distributed under the terms of the Creative Commons Attribution License (CC BY 4.0), which permits unrestricted use, distribution, and reproduction in any medium, provided the original author and source are credited.Peter Ustjuzhanin, Vasily Kovtunovich, Szabolcs Sáfián, Vincent Maicher, Robert Tropek
